# Microbial Musings – January 2020

**DOI:** 10.1099/mic.0.000882

**Published:** 2020-01-21

**Authors:** Gavin H. Thomas

**Affiliations:** ^1^​ Department of Biology, University of York, York, YO10 5YW, UK

**Keywords:** *Pseudomonas*, *Staphylococcus*, *Saccharomyces cerevisiae*

Ever since I read John Postgate’s *Microbes and Man* when I was at school I have been fascinated by the microbial world. Microbes are so amazing in what they can do and how they have evolved to do it that understanding even a fraction of their potential will keep many generations of scientists busy for decades to come. As a working microbiologist, Twitter is a great way to keep up to date with exciting new papers and the almost daily ‘wow’ when I see some new piece of excellent science is a good way to start the day – if only there was time to read but a fraction of them! As Twitter and other social media formats have replaced ‘old school’ traditions of browsing a physical journal issue with a printed table of contents, we now sometimes miss that important but slightly tangential paper that could inform our own research – the power of reading a serendipitous abstract is strong. In an attempt to bring this back in a virtual sense, we bring you Microbial Musings, a monthly digest of selected papers published in *Microbiology* with (hopefully) useful links to people, places and other Microbiology Society activities, starting with the first issue of 2020.

We start the first musings with a cluster of papers on the genus *Pseudomonas *, with the most (in)famous species *Pseudomonas aeruginosa* the focus of our latest Microbe Profile. When I was learning microbiology in the early 1990s, we were told that this bug was mainly associated with infections of severe burns and as such was a textbook opportunistic pathogen. This Microbe Profile, written by *Microbiology* Editor Stephen Diggle (@Digglelab) and recent Senior Editor Marvin Whiteley (@Whiteleylab), both at Georgia Institute of Technology, Atlanta, GA, USA, provides an excellent update on this pathogen that highlights key features of its biology, including many new studies of how it has adapted to colonize the lungs of people living with the genetic condition cystic fibrosis (CF) [1]. There is lots of great information captured in the article – for instance, I never knew that some strains can display two distinct types of lipopolysaccharide (LPS) on the cell surface at the same time. One feature of *P. aeruginosa* that has always intrigued, or to be honest slightly scared, me, is the complexity of the regulation of its quorum sensing (QS) pathways. As Diggle and Whiteley point out, quorum sensing in *P. aeruginosa* has been developed as a model system for studying social evolution theory. Another paper in this issue takes this further by looking at the interaction of one of the known QS molecules, the *Pseudomonas* quinolone signal (PQS), a small molecule that is also known to be able to chelate iron [2]. The researchers from David Steven’s group at Stanford, CA, USA, investigate how bacterial-derived PQS can modulate the function of the fungus *Aspergillus fumigatus*, a fungal pathogen that can co-infect the CF lung. At low Fe^3+^ concentrations the presence of PQS inhibits fungal growth through iron limitation, which is reversed by adding more iron. Interestingly, at high iron concentrations PQS actually enhances *A. fumigatus* growth, possibly through stimulating its own siderophores. Hence, the interaction of these two microbes is highly environmentally regulated and highlights one of Diggle and Whiteley’s open questions for *P. aeruginosa *, namely ‘How does spatial structure impact on interactions between *P. aeruginosa* strains and other species of microbes?’

While *P. aerugonisa* often takes the headlines, the biological diversity of this genus is notably broad, from soil strains with important roles in biotechnology such as *Pseudomonas putida* to well-studied pathogens of plants such as *Pseudomonas fluorescens* and others. *Microbiology* editor Vittorio Venturi and his group from the ICGEB Trieste, Italy (@ICGEB), working with David Studholme at Exeter (@davidjstudholme), have been studying the biological fate of azeleic acid, a plant-derived long-chain (C9) dicarboxylic acid that has been implicated in plant–bacterial signalling [3]. Perhaps not surprisingly, other soil bacteria appear to be able to eat this carbon and energy source, including the soil bacterium *Pseudomonas nitroreducens *, in a process first described in 1964 in a paper in our journal when it was still known as the *Journal of General Microbiology* [4]. Using a genetic approach, the paper describes the identification of a gene cluster required for azeleic acid utilization, which uses a pathway that appears similar to the beta-oxidation routes used by bugs to eat fatty acids. In the cluster they identify a new transcription factor, which they call AzeR, and they present data consistent with this being a transcriptional repressor that is activated by azeleic acid.

Next we switch to *P.* *fluorescens* *﻿*, the organism chosen in another interesting study from the group of Mike Brockhurst and colleagues in Sheffield, including Rosanna Wright (@rctwright) and Ellie Harrison (@ellieevolves). The first author, Jamie Hall (@jpjhall), now a new lecturer at the University of Liverpool, studied the rapid evolution of compensatory chromosomal mutations to reduce the burden of carrying a plasmid [5]. In this particular study Jamie used a plasmid with an initially very high fitness cost, so much so that it caused a small colony phenotype on plates. Mutants were isolated relatively rapidly that restored the normal colony size, while maintaining plasmid function. Multiple evolutionary trajectories led to a number of different chromosomal allelles that enabled reduced plasmid cost, including the GacAS two-component system – one of the components of the highly complex regulatory networks also seen in *P. aeruginosa,* where altered levels of RsmA, a small translational regulator, are also known to result in the emergence of small colony variants in *P. aeruginosa* samples isolated in the CF lung [6]. Other trajectories result in single-nucleotide polymorphisms (SNPs) within a DNA-binding protein of unknown function, revealing new clues to gene functions that could limit plasmid cost. Catch both Jamie (chairing) and Ellie (speaking) at the ‘Secret life of mobile genetic elements’ symposium at Annual Conference 2020!

The concept of interdomain interactions between microbes, previously highlighted between *P. aeruginosa* and *A. fumigatus*, takes another angle in new work from Jaelle Brealey (@JaeBrealey), derived from her PhD work in the groups of Paul Young (@ProfPaulYoung) and Keith Chappell (@ChappellDr) at the University of Queensland, Adelaide, Australia [7]. She has been studying another kind of interaction between microbes, in this case between the bacterium *Streptococcus pneumoniae* and respiratory syncytial virus (RSV). In her previous work, she and others demonstrated a significant association between the presence of RSV and *S. pneumoniae* in children with acute respiratory infection. In this work, the genetic variations of the protagonists are examined, revealing that no particular strains of RSV or *S. pneumoniae* are found together. The proposed mechanism of their co-infection is via the binding of the bacterium to host cell surface molecules upregulated by RSV infection and also direct binding to RSV surface glycoproteins on the virus surface, which suggests that these interactions are common to all the tested bacterial strains, rather than being strain- or serotype-specific.

Finally, a couple of articles that relate to how microbes take up and export small molecules, a subject close to my heart. First, Ashfaq Ahmad and colleagues at Hazara University, Pakistan review new literature on the interplay between two-component systems and efflux pumps in the mechanisms of resistance to a range of different peptide-based antibiotics [8]. Many examples are provided across a range of pathogens, and a subset of ABC transporters involved in efflux have an additional protein sequence that appears to function directly in binding the substrates or ‘talking’ to the matched two-component system in the membrane to indicate the presence of substrate. The review highlights much work, including that of *Microbiology* editor Susanne Gebhard (@GebhardLab) at the University of Bath, UK, who recently published on one of these components, BcrR, in the journal [9], and recent *Microbiology* senior editor Tarek Msadek at the Pasteur Institute, Paris, who has published extensively on the systems of this type that operate in *Staphylococcus aureus *.

The final paper from the selection studies the complex regulation of a branched-chain amino acid transporter from the yeast *Saccharomyces cerevisiae* called BAP2, from the lab of Mariana Bermudez-Moretti in Buenos Aires, Argentina [10]. Expression of the gene is induced when non-preferred nitrogen sources are provided and in this paper the authors identify the cross-regulation of *BAPA* by a known transcription factor called Uga3, which is involved in γ-aminobutyric acid (GABA)-dependent induction of the GABA genes. As GABA is also another alternative nitrogen source that *S. cerevisiae* can use, this suggests a wider function for Uga3 in coordinating gene expression in response to nitrogen limitation in this model eukaryotic microbe.

So, that ends the first musings. As we enter a new decade of *Microbiology*, we can look forward to constantly deepening our understanding of the microbial world and the important role it can play in addressing many challenges that we face – look out for the Microbiology Society’s initiative on sustainability and ‘Why Microbiology Matters’, which launch in 2020, and join us in Edinburgh for the Microbiology Society’s 75th Annual Meeting over a bumper 5 days of springtime microbes.

Gavin Thomas, Deputy Editor-in-Chief.
